# Culturally Adapted, Clinician-Led, Bilingual Group Exercise Program for Older Migrant Adults: Single-Arm Pre–Post-Intervention

**DOI:** 10.3390/ijerph22060888

**Published:** 2025-06-02

**Authors:** Morwenna Kirwan, Christine L. Chiu, Jonathon Fermanis, Katie Allison, Thomas Laing, Kylie Gwynne

**Affiliations:** 1 Nura Gili: Centre for Indigenous Programs, University of New South Wales, Kensington, NSW 2052, Australia; kylie.gwynne@unsw.edu.au; 2 Faculty of Medicine, Health & Human Sciences, Macquarie University, North Ryde, NSW 2109, Australia; christine.chiu@mq.edu.au; 3 Diabetes Australia, Glebe, NSW 2037, Australia

**Keywords:** community-based, culturally adapted, mental health, physical fitness, older adult, clinician led, migrant, group exercise, exercise physiologist, health promotion

## Abstract

Australia’s aging migrant population experiences higher rates of chronic disease and social isolation, highlighting the need for culturally appropriate health promotion programs. This study evaluated the effectiveness of “Move Together”, a culturally adapted community-based group exercise and education intervention for Mandarin-speaking Chinese Australians aged 65+ years. The Model for Adaptation, Design, and Impact framework guided the adaptation of this program. This single-arm pre–post-intervention was delivered bilingually by accredited exercise physiologists over nine weeks to 101 adults (mean age: 72.3 ± 5.3 years; 38% male). The physical health assessments measured waist circumference, aerobic capacity, strength, flexibility, and balance, while the questionnaires evaluated quality of life and social connectedness. The results showed significant improvements in all physical health measures (*p* < 0.001), with more participants meeting fitness standards for healthy independent living. Participants also reported significant improvements in self-perceived quality of life and social connectedness. The Move Together program effectively improved physical health and psychosocial well-being among older Chinese Australians through its culturally adapted, bilingual delivery model. These findings provide valuable insights for health professionals and policymakers adapting and implementing programs for culturally and linguistically diverse older populations to address chronic disease risks and social isolation.

## 1. Introduction

Over one-third of Australia’s population is born overseas, with Chinese Australians representing one of the largest ethnic minority groups [1]. Mandarin now ranks as the most commonly spoken language at home after English [2]. This demographic faces elevated risks of chronic diseases alongside greater social isolation and loneliness [3,4], creating an urgent need to address the physical and mental health needs of aging migrant populations [5]. These elevated risks arise from multiple factors, including cultural and language barriers to healthcare access, dietary transitions, and reduced physical activity participation due to cultural adaptation challenges [4].

Exercise offers substantial physiological and psychological benefits for older adults [6] and serves as the cornerstone of comprehensive chronic disease management [7]. Regular exercise in older adults has been shown to reduce the risk of falls [8], osteoporosis [9], and muscular atrophy [10] while improving sleep quality [11] and blood pressure [12]. Exercise also has been shown to be beneficial for slowing cognitive decline [13] and functional disability [14,15] and improving cardio-metabolic health [16], mood and mental health [17,18], and social connections [19]. Despite these benefits, one-third of adults remain insufficiently active [20], with even lower participation rates among culturally and linguistically diverse (CALD) populations [4,21,22]. Within CALD communities, lower participation may be attributed to cultural differences, language barriers, and lower levels of health literacy [23], which make accessing health programs challenging [24,25]. This problem is further exacerbated by the scarcity of physical activity and exercise initiatives tailored to meet the specific needs of these communities [26,27].

Clinician-led, community-based group exercise interventions are recognized as effective strategies to improve the physical and mental health and well-being of older adults [4,18,28,29,30,31,32]. For CALD populations, adapting such programs offers dual benefits: enhanced health outcomes and strengthened social connectedness. Despite this potential, a substantial knowledge gap exists regarding best practices for adapting exercise interventions for CALD communities [26,33,34,35]. Successful cultural adaptation requires addressing both surface-level factors (such as language) and deeper structural elements (including cultural norms, social contexts, historical influences, and psychological factors that shape health behaviors within specific CALD communities) [36,37]. Addressing these multifaceted factors is crucial to ensure CALD communities have access to and can benefit from these programs.

The Beat It program is a national, clinician-led, group exercise program that has been shown to improve physical fitness, waist circumference, and self-reported quality of life in older Australians managing a chronic disease [29,30,31]. Building on this evidence, Move Together was developed as a culturally adapted version specifically for Mandarin-speaking Chinese Australian adults. This tailored program was designed to address barriers faced by older Chinese Australian adults in accessing community health services while fostering social connectedness. This study aimed to evaluate Move Together’s effectiveness in improving physical health, fitness outcomes, social connectedness, and quality of life among older Chinese Australians.

## 2. Materials and Methods

We adhered to the Strengthening the Reporting of Observational Studies in Epidemiology (STROBE) guidelines [38] throughout this manuscript (Supplementary File S1) and the Template for Intervention Description and Replication (TIDieR) checklist [39] for comprehensive intervention reporting (Supplementary File S2).

### 2.1. Program Adaptation Framework

The Model for Adaptation, Design, and Impact (MADI) [40] framework guided the adaptation of the Beat It Program into Move Together. MADI has three domains: adaption characteristics (what was modified and how, for whom, and when, and who was involved in decision making); moderating or mediating factors (adaptation aligned with the core elements of the intervention or implementation strategy, clear goals for adaptation, and adaptations implemented consistently); and intended and unintended outcomes. Analysis across these three domains helps explain the impact [40].

### 2.2. Intervention Description

The Move Together program was a nine-week intervention consisting of one supervised group exercise session per week, complemented by education sessions. The moderate-intensity group exercise sessions were supervised by bilingual (Mandarin- and English-speaking) accredited exercise physiologists (AEPs) in New South Wales (NSW). AEPs are university-qualified exercise professionals who follow Exercise and Sports Science Australia (ESSA) Standards for Exercise Assessment and Prescription, which provide the foundation for clinical practice. All AEPs completed a specialized 14-hour online facilitator training program (accredited as continuing professional development) to ensure consistent and effective delivery. Group sessions were limited to 12 participants to ensure adequate supervision and support.

Prior to beginning the program, participants had a one-on-one consultation with an AEP to assess their physical capabilities, fitness level, and any co-morbidities or injuries that may impact their ability to participate. The AEP then developed a personalized exercise program, including a dynamic warm-up and cool-down, aerobic, resistance, balance, and flexibility exercises. Throughout the intervention, the AEP adjusted exercises according to participants’ progress. Group education sessions were held four times over the nine weeks and focused on chronic disease prevention through physical activity, healthy eating, and mental health management.

### 2.3. Participants and Recruitment

Participants were recruited between February and September 2022 through targeted ethno-specific channels, including email campaigns, radio broadcasts, website advertisements, print marketing materials, and community outreach. Recruitment efforts were tailored to engage the Chinese community, leveraging connections with community leaders and Chinese community groups. The inclusion criteria were as follows: (1) 65 years or older, (2) Mandarin-speaking, and (3) medical clearance from their general practitioner to exercise. Participants underwent an initial health and fitness assessment with their designated AEP, and a final health and fitness assessment was conducted on completion of the program. All program delivery followed COVID-19 safety protocols in accordance with NSW Health guidelines, current at the time of implementation.

Only participants who were 65 years or older and who completed the initial and final health and fitness assessments were included in this study. The Macquarie University Human Ethics Committee approved the study under protocol number 5201950887424.

### 2.4. Measures and Assessment

This study utilized a pre–post-evaluation design, with individual physical assessments conducted at baseline and at nine weeks following the completion of the Move Together program. The sociodemographic variables recorded included gender and date of birth. Height and weight were measured to calculate body mass index (BMI), categorized as underweight, healthy, overweight, or obese according to the Asian guidelines set by the World Health Organization [41]. Waist circumference was classified as normal or at risk based on criteria for Asian males (<90 cm) and females (<80 cm) [42]. Upper and lower body strength, aerobic capacity, balance, and flexibility were assessed using the 30-s arm curl test, the 30-s sit-to-stand test, the six-minute walk test, the timed one-legged stand test, and the chair sit-and-reach test, respectively [43]. These assessments are components of the Senior Fitness Test, developed by Rikli and Jones [44], a validated battery of functional fitness tests specifically designed for older adults. These standardized assessments were selected based on their established validity and reliability for measuring functional fitness in older adults and their direct relevance to daily living activities that support independent living. The Senior Fitness Test provides age-appropriate measures that correspond to physical abilities needed for maintaining mobility and functional independence in later life [44]. Participants with injuries, recent surgery, or other physical limitations were excluded from tests that were contraindicated or posed additional risk. Baseline and post-program fitness measures were dichotomized as shown below or meeting the fitness standard based on criterion-referenced fitness standards for older Chinese adults [45].

At both baseline and program completion, participants completed a questionnaire to assess their motivations for joining and evaluate the program’s impact on quality of life, health behaviors, social inclusion, and mental health using a five-point Likert scale. This evaluation instrument, developed by Diabetes Australia specifically for program evaluation and reporting to the funding body, is not a validated assessment tool (Supplementary File S3).

### 2.5. Data Analysis

Data analysis was performed using SPSS version 27 (SPSS Inc., Chicago, IL, USA). Means and standard deviations (SDs) were reported for continuous variables, and frequencies and percentages were reported for categorical variables. Participants with missing data for any pre- or post-program fitness measure were excluded from the analysis. The effectiveness of the Move Together program in anthropometric and physical fitness measures was assessed using paired t-tests, stratified by gender, for continuous variables. For dichotomous variables, the paired-sample McNemar test was used to compare pre- and post-program effects, stratified by gender. A Bonferroni-corrected p-value of less than 0.001 was considered significant to account for multiple tests.

## 3. Results

### 3.1. Move Together MADI Results

Nine clinical, practical, and technical aspects of Beat It were adapted for the Move Together program (Table 1). Each aspect included moderating and mediating factors to support program fidelity. The intended outcomes were improvements in functional fitness and physical health across demographics (Table 2). The unintended outcomes are currently unknown and will be explored in a follow-up study. Overall, the Move Together program was as effective as the in-person Beat It program [30].

### 3.2. Move Together Participants’ Results

**Figure 1:**
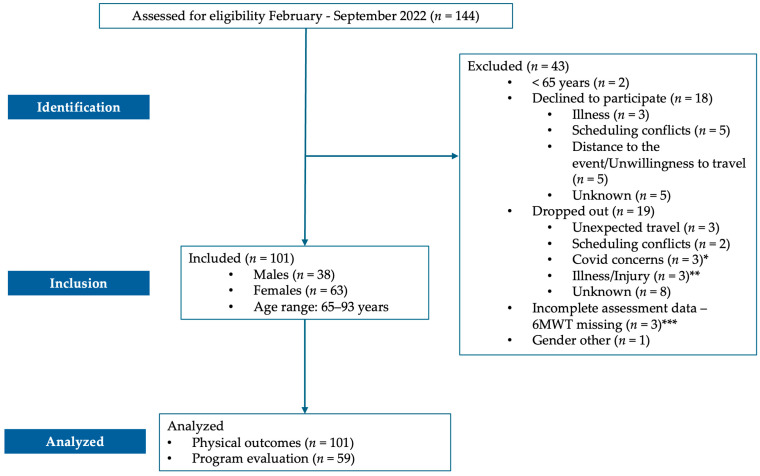
Flow diagram of participant recruitment, inclusion, and analysis for the Move Together program. * Participants did not have COVID-19 but were fearful of exposure. ** One participant got COVID-19 and dropped out, and two had falls not related to the program. *** 6MWT—six-minute walk test.

Out of 144 individuals assessed for eligibility, 43 were excluded: 2 were under 65 years of age, 18 declined to participate, 19 dropped out, 3 had incomplete final assessment data, and 1 reported gender as “other”, which could not be accommodated in the gender-stratified analysis (Figure 1).

The 101 participants completed the Move Together program at four sites (Chatswood, Hurstville, Petersham, and Rhodes) in NSW, delivered by six bilingual AEPs. Participants’ ages ranged from 65 to 93 years (mean: 72.3 ± 5.3 years). Male participants (*n* = 38, 38%) had a mean age of 71.6 ± 5.0 (range: 65 to 86 years), while female participants (*n* = 63, 62%) had a mean age of 72.7 ± 5.5 years (range: 65 to 93 years).

Participants attended between three and nine exercise sessions, with 84% attending at least seven of the nine sessions. For the education sessions, participants attended between one and four sessions, with 88% attending at least three of the four education sessions.

At the beginning of the program, 77% of males and 48% of females were overweight or obese based on BMI [41]. In addition, over two-thirds of participants (71% males and 76% females) had a waist circumference indicating an increased risk of chronic disease [42]. Baseline fitness assessments showed many participants performed below the standard for healthy independent living for their gender and age, taking into account ethnicity [45].

**Figure 2:**
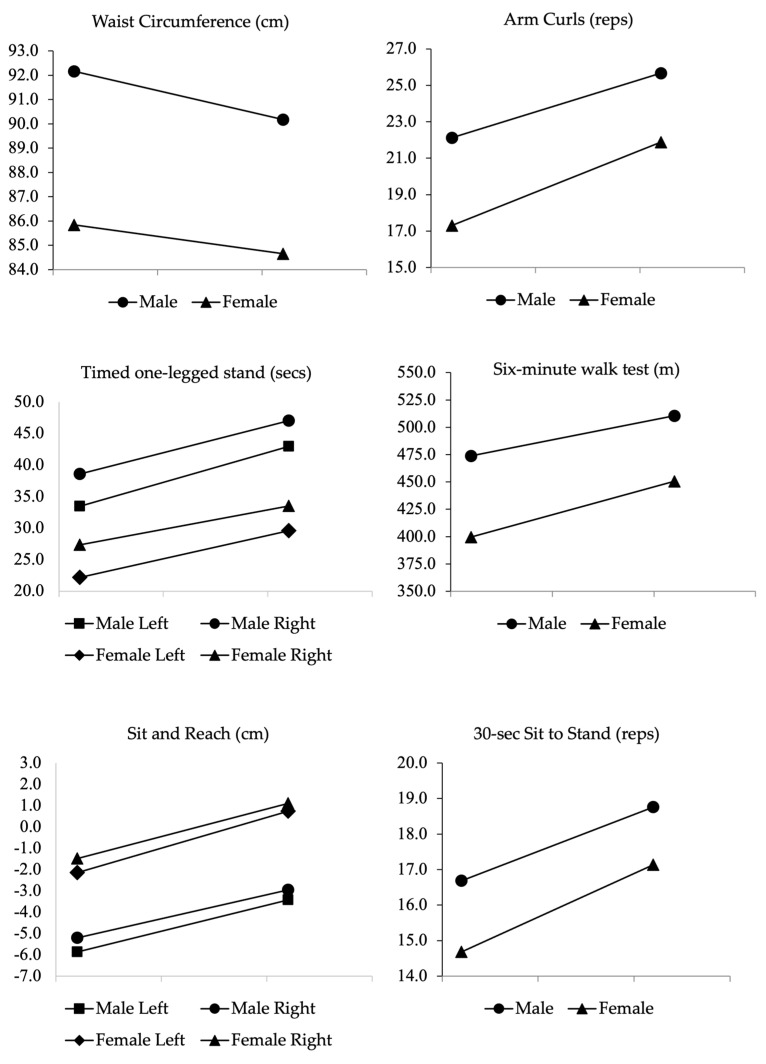
Anthropometric and fitness measures at baseline and post-intervention (nine weeks). Units of measurement: cm = centimeters; reps = repetitions; secs = seconds; m = meters.

Following the 9-week intervention, significant improvements were observed in waist circumference, aerobic capacity, strength, flexibility, and balance for both male and female participants (Table 2 and Figure 2). The number of participants meeting fitness standards for healthy independent living also increased post-program (Table 3).

Participants evaluated the program’s impact on their quality of life, health behaviors, and mental health through pre- and post-program evaluation questionnaires. Of the 101 participants, 59 (54% female) completed both the pre- and post-evaluation questionnaires. The most common reason for joining the program was to improve health (92%). Post-program, 90% reported increased motivation to look after their health, and 100% found the education sessions useful. Participants agreed that the program was easy to understand (100%), suited their needs (98%), offered a supportive environment (92%), and made them feel more connected to a supportive community (88%). Initially, 78% rated their quality of life as good, which increased to 91.5%, rating it as excellent post-program.

## 4. Discussion

This study demonstrated that a culturally adapted, clinician-led, bilingual, supervised group exercise and education program benefits the health of older Chinese Australians. Interventions like Move Together are important for practitioners and policymakers seeking to provide culturally responsive care that maintains the independence of older adults, mitigates frailty, strengthens social connections, and improves functional and physical fitness and quality of life. These findings are particularly relevant, as Chinese Australians experience higher levels of physical inactivity compared with their non-Chinese counterparts [46]. They are also at increased risk of noncommunicable diseases like cardiovascular disease and type 2 diabetes [4], age-related functional decline, which raises susceptibility to falls and frailty [47], and elevated levels of loneliness and social isolation [3,48,49].

The effectiveness of Move Together may be attributed to its considered cultural adaptation. Integrative [35], scoping [50], and systematic reviews [33,51] have highlighted important components of effective physical activity programs for CALD older adults. These components include accessible community venues; the involvement of the community in program design; a collectivist rather than individualistic approach; social opportunities for peer engagement; language adaptation of materials; and the incorporation of culturally familiar activities [33,35,50,51]. The Move Together program incorporates all of these elements, emphasizing geographical accessibility, cultural relevance, effective communication, group interactions, and a sense of belonging via familiar activities. An additional strength was the use of bilingual health professionals who shared the cultural backgrounds of participants, a factor previously recognized as highly effective in similar interventions [21,32,33,35,52]. The results of Move Together align with two clinician-led group exercise interventions for older Chinese immigrants in the United States [32,52], which also reported high completion rates and positive participant feedback.

The Move Together program also incorporated evidence-based behavioral change techniques (BCTs) [53], which are known to be effective in improving health and well-being in older migrant populations [54]. Techniques included goal setting and planning (using problem solving and behavioral contracts), social support, knowledge enhancement (specifically, guidance on exercise performance), repetition and substitution (e.g., practicing target behaviors in various contexts), the recognition of natural consequences (education about health, social, and environmental consequences), and behavior comparison (utilizing social comparisons). Further research is needed to understand the interplay of these BCTs within culturally adapted programs and to elucidate the mechanisms through which they influence behavioral change. This could enhance our understanding of intervention effects.

This study utilized MADI, an implementation science framework, to evaluate and report on adaptations made to the Beat It program [40]. Key adaptation characteristics, their intended and unintended impacts, and potential mediators and moderators of outcomes were identified. The comprehensive reporting of modifications is crucial, as research suggests that many interventions adapted for CALD populations lack transparency in their cultural adaptations and are often limited to language translations of materials [26,55,56].

A notable strength of the Move Together program lies in its broad implementation across multiple sites, with diverse bilingual health professionals. However, some limitations should be noted. Firstly, this study employed a pre–post-evaluation without a comparison group, a method commonly used in translational community-based programs [57]. Secondly, the pre- and post-survey questions were developed specifically to evaluate this program and have not been validated as standardized assessment tools. Furthermore, the evaluation focused on the short-term impact of Move Together. Future studies that include a longer follow-up period are warranted to assess whether participants maintain the program’s benefits over time. For reference, a follow-up study of the Beat It program showed participants sustained health improvements 12 months after completing the program [31].

## 5. Conclusions

The aging migrant population highlights the pressing need for culturally responsive translational health programs and services. Move Together offers a culturally safe and scalable solution that provides significant physical and mental health benefits. Long-term evaluation of this program is needed to assess its continuing impact on health outcomes.

## Figures and Tables

**Table 1:** MADI report of adaptations and mediating and moderating factors.

Adaptation Areas	Mediating/Moderating Factors	
Program	Beat It (In Person)	Move Together
Facilitator training	12 h of online learning; 1-day in-person practical training	Additional 2 h of online training covering social isolation and inclusion, Move Together program logistics, facilitator requirements, and key considerations
Marketing	Direct mail (via post), email, and website (English only)	Digital—email and website, print marketing (English and simplified Chinese), radio advertising, and community outreach via new and established links with community leaders and Chinese community groups
Participant resources	Beat It participant handbook Home exercise resource Theraband	All participant-facing resources are culturally adapted and translated by Mandarin-speaking health professional staff of Chinese background and translated into simplified Chinese (written Mandarin script) through an external translation company with community review of translated materials included to ensure accuracy and cultural appropriateness Move Together participant handbook Education session topics altered to suit general population attendees rather than diabetes-specific information Removal of diabetes-specific content e.g., blood glucose level logs Simplified-Chinese-translated Guide to Healthy Eating brochure Theraband
Trainer resources	Beat It in-person delivery manual Beat It facilitator manual (education sessions)	Move Together delivery manual adapted from Beat It Additional information on the purpose of the program and ways to promote social inclusion Adjustment to program inclusion criteria to suit > 65-year-old Mandarin-speaking participants Changes to program timeline and requirements Adapted Move Together facilitator manual Changes to content to reflect educational topics Inclusion of language-specific and culturally adapted resources to use during education and exercise sessions
Medical clearance	Standardized medical clearance form, including recommended program inclusion/exclusion criteria, medical history, medications, and latest HbA1c and lipid test results Participants typically bring a physical copy of a medical clearance form to the initial consultation with the Beat It trainer	Additional considerations and exclusion criteria for determining suitability to join the Move Together program including 65 years or older (requirement of funding to tailor the program for people over 65 years of age); Mandarin-speaking person
Pre-program	Pre-program resources sent including a welcome letter confirming program registration, medical clearance, and initial consultation process Beat It trainer books initial assessment appointment	Participant materials sent translated into simplified Chinese Confirmation letter Medical clearance Schedule template Move Together trainer books initial assessment
Initial and final assessment	Conducted in person Obtain medical clearance, participant informed consent, and emergency contact information and complete pre-screening questionnaire Complete baseline measurements, including height, weight, waist circumference, BP, and HR Complete exercise tests, including 6-min walk test (6MWT), 30-s sit-to-stand, 30-s seated arm curl test, seated sit and reach, and single-leg stance test Goal setting	Conducted in person in Mandarin (or preferred language relative to the individual) Initial assessment normative data adapted to Asian population group in testing protocols resource Complete pre- and post-evaluation data outlining impact of program on social inclusion
Exercise sessions	Capped at 12 participants per session In-person exercise sessions consist of a warm-up, followed by a combination of aerobic, resistance, balance, and flexibility exercises tailored to participants’ abilities, followed by a cool-down period	Capped at 12 participants per session delivered in Mandarin Trainers encouraged to factor social inclusion into structure of sessions, e.g., group activities, “pairing” participants together, the general promotion of conversation, and relationship building
Education sessions	6 × 30 min person-centered education sessions on various lifestyle and diabetes management topics delivered in person	4 × 30 min person-centered education sessions on various lifestyle management topics delivered in person in Mandarin Removal of diabetes-specific information and topics to ensure suitability to general population Focus on prevention of conditions like diabetes through physical activity, healthy eating, and mental health management

Note. BP = blood pressure; HR = heart rate; HbA1c = glycated hemoglobin.

**Table 2:** Anthropometric and fitness measures at baseline and post-intervention.

	Male (*n* = 38)			Female (*n* = 63)		
	Baseline Mean (SD)	9-Week Mean (SD)	99% CI; *p*-Value	Baseline Mean (SD)	9-Week Mean (SD)	99% CI; *p*-Value
Weight (kg)	70.7 (8.2)	70.6 (8.1)	[−0.41–0.60]; 0.61	56.2 (7.0)	56.04 (6.9)	[−0.16–0.47]; 0.19
BMI (kg/m^2^)	24.71 (2.0)	24.68 (1.9)	[−0.14–0.21]; 0.61	23.05 (3.0)	22.99 (3.0)	[−0.07–0.2]; 0.19
Waist circumference (cm)	92.17 (6.0)	90.18 (6.0)	[0.23–3.75]; 0.004	85.85 (8.7)	84.66 (8.5)	[0.47–1.9]; <0.001
Sit and reach–left (cm)	−5.87 (9.1)	−3.42 (8.9)	[−3.96–-0.93]; <0.001	−2.14 (8.9)	0.74 (8.0)	[−4.08–−1.68]; <0.001
Sit and reach–right (cm)	−5.2 (8.6)	−2.95 (8.2)	[−3.87–−0.63]; <0.001	−1.49 (9.0)	1.1 (8.3)	[−3.77–−1.42]; <0.001
30 sec sit to stand (reps)	16.68 (4.8)	18.76 (4.4)	[−3.47–−0.68]; <0.001	14.68 (4.7)	17.13 (4.8)	[−3.32–−1.57]; <0.001
One-legged stand–L (sec)	33.45 (22.0)	42.92 (21.6)	[−15.62–−3.33]; <0.001	22.16 (17.9)	29.56 (20.7)	[−11.89–−2.90]; <0.001
One-legged stand–R (sec)	38.55 (22.3)	47.03 (20.6)	[−14.78–−2.17]; <0.001	27.32 (22.2)	33.48 (20.5)	[−9.89–−2.42]; <0.001
Arm curl (reps)	22.13 (4.8)	25.66 (5.4)	[−5.43–−1.63]; <0.001	17.3 (5.4)	21.89 (5.4)	[−5.83–−3.35]; <0.001
Six-minute walk distance (m)	474 (130.9)	510.66 (128.2)	[−72.69–−0.63]; 0.01	399.54 (117.7)	450.63 (138.5)	[−74.29–−27.9]; <0.001

Note. CI = confidence interval; BMI = body mass index; kg = kilogram; m = meter; cm = centimeter; sec = seconds; reps = repetitions; L = left; R = right; SD = standard deviation.

**Table 3:** Proportions of participants who met or were above the standard [45] for anthropometric and fitness measures.

	Male (*n* = 38)			Female (*n* = 63)		
	Baseline Count (%)	9-Week Count (%)	*p*-Value	Baseline Count (%)	9-Week Count (%)	*p*-Value
Body mass index						
Normal (18.5–22.9)	9 (24)	9 (24)		33 (52)	33 (52)	
Overweight (23.0–24.9)	13 (34)	13 (34)		17 (27)	17 (27)	
Class I obesity (25–29.9)	15 (39)	15 (39)		11 (18)	11 (18)	
Class II obesity (≥30)	1 (3)	1 (3)		2 (3)	2 (3)	
Waist circumference (cm)						
Normal range	11 (29)	16 (42)		15 (24)	17 (27)	
Risk of chronic disease	27 (71)	22 (58)	0.06	48 (76)	46 (73)	0.50
Sit and reach (cm)						
Below standard	22 (58)	19 (50)		48 (76)	43 (68)	
Met or were above standard	16 (42)	19 (50)	0.25	15 (24)	20 (32)	0.18
30 sec sit to stand (reps)						
Below standard	15 (40)	6 (16)		32 (51)	19 (30)	
Met or above standard	23 (60)	32 (84)	0.12	31 (49)	44 (70)	<0.001
30 sec arm curl (reps)						
Below standard	2 (5)	0 (0)		23 (37)	9 (14)	
Met or above standard	36 (95)	38 (100)		40 (63)	54 (86)	<0.001

Note. cm = centimeters; reps = repetitions; sec = seconds. *p*-values not reported for BMI categories due to no participants changing categories between time points. *p*-value not reported for male 30-s arm curl due to small sample size (*n* = 2) initially below standard.

## Data Availability

The data that support the findings of this study are available upon request from the corresponding author, Morwenna Kirwan.

## Supplementary Materials

The following supporting information can be downloaded at https://www.mdpi.com/article/10.3390/ijerph22060888/s1. File S1: STROBE guidelines checklist; File S2: Template for Intervention Description and Replication (TIDieR) checklist; File S3: Diabetes Australia Pre–Post-Survey Questions.
