# Supplementary material for: Culturally Adapted, Clinician-Led, Bilingual Group Exercise Program for Older Migrant Adults: Single-Arm Pre–Post-Intervention
Source: Int J Environ Res Public Health. 2025 Jun 2;22(6):888. doi: 10.3390/ijerph22060888 (PMC12193282; doi:10.3390/ijerph22060888)
Supplement: Supplementary file 1 [file ijerph-22-00888-s001.zip › Move Together - Supplemental File 3 - Pre and Post Program Survey.pdf]

Move Together
(Pre-Program Survey)

Welcome to Move Together. Before we begin, we would appreciate if you could take a few minutes to complete this survey for research and quality improvement purposes. Your responses and any identifying information will be kept confidential.

Today's date: DD/MM/YYYY (DD/MM/YYYY) **Program location (suburb):** ___________________

Date of Birth: (DD/MM/YYYY)

D	D	M	M	Y	Y	Y	Y

What is your FIRST name (Given Name)**?** ______________________________

Gender: ☐ Male ☐ Female ☐ Other

What is your postcode (of your home address)**?** ☐☐☐☐

In general, would you say your present quality of life is:

Excellent	Very Good	Good	Neither good nor bad	Bad	Very bad	Extremely bad

In general, would you say your health is:

1 Excellent 2 Very Good 3 Good 4 Fair 5 Poor

What has motivated you to join Move Together?

- ▢ It is free
- ▢ I want to improve my health
- ▢ It is delivered in Mandarin
- ▢ I want to meet new people from my community
- ▢ It is close to home
- ▢ Other, please specify: ____

Please indicate for each of the five statements which is closest to how you have been feeling over the last two weeks. Notice that answers closer to the left mean better well-being.

Example: If you have felt cheerful and in good spirits more than half of the time during the last two weeks, you would choose the 3rd option from the left.

Over the last two weeks:	All the time	Most of the time	More than half of the time	Less than half of the time	Some of the time	At no time
1. I have felt cheerful and in good spirits	5	4	3	2	1	0
2. I have felt calm and relaxed	5	4	3	2	1	0
3. I have felt active and vigorous	5	4	3	2	1	0
4. I woke up feeling fresh and rested	5	4	3	2	1	0
5. My daily life has been filled with things that interest me	5	4	3	2	1	0
6. I have felt connected with my community	5	4	3	2	1	0

Thank you for taking the time to complete this survey, we hope you enjoy the program.

DNSW & ACT MAY OCCASIONALLY USE SURVEY COMMENTS FOR PROMOTIONAL MATERIALS SO THAT OTHERS MAY BETTER UNDERSTAND THE BENEFITS OF ATTENDING. INFORMATION COLLECTED WITHIN THIS SURVEY IS COVERED BY THE DNSW & ACT PRIVACY POLICY. FOR MORE INFORMATION VISIT DIABETESNSW.COM.AU/PRIVACY/ OR CALL 1300 342 238.

Move Together (Post-Program Survey)

Thank you for attending Move Together, we hope you enjoyed it. We would appreciate if you could spend five minutes or so to complete this survey. Your responses and any identifying information will remain confidential. Some of the questions in this survey will be the same as the ones you completed before the program. This allows us to monitor changes in your responses over time.

Today's date: DD/MM/YYYY (DD/MM/YYYY) **Program location (suburb):** ________________

Facilitator ID (please ask your facilitator for this) ________________________

Date of Birth: (DD/MM/YYYY) D D M M Y Y Y Y

What is your FIRST name (Given Name)**?** ____________________________

Gender: ☐ Male ☐ Female ☐ Other

What is your postcode (of your home address)**?** ☐☐☐☐

In general, would you say your present quality of life is:

Excellent	Very Good	Good	Neither good nor bad	Bad	Very bad	Extremely bad

In general, would you say your health is:

1 Excellent 2 Very Good 3 Good 4 Fair 5 Poor

**Please choose one option for each statement below**	Not at all	Very little	To some extent	To a great extent	100% extent	Don't know
1. To what extent are you proud to be part of the Move Together group?	1	2	3	4	5	6
2. To what extent do you take pride in your group's achievements?	1	2	3	4	5	6

diabetes nsw & act

Please indicate for each of the five statements which is closest to how you have been feeling over the last two weeks. Notice that answers closer to the left mean better well-being.

Example: If you have felt cheerful and in good spirits more than half of the time during the last two weeks, you would choose the 3rd option from the left.

**Over the last two weeks:**	All the time	Most of the time	More than half of the time	Less than half of the time	Some of the time	At no time
1. I have felt cheerful and in good spirits	5	4	3	2	1	0
2. I have felt calm and relaxed	5	4	3	2	1	0
3. I have felt active and vigorous	5	4	3	2	1	0
4. I woke up feeling fresh and rested	5	4	3	2	1	0
5. My daily life has been filled with things that interest me	5	4	3	2	1	0
6. I have felt connected with my community	5	4	3	2	1	0

Please let us know how much you agree or disagree with the below statements.

**Please choose one option for each statement:**<br><br>**Since attending the program:**	Disagree ☹	Neutral 😐	Agree ☺	Don't know
1. Meeting and engaging with new people as part of the program was a pleasant experience	1	2	3	4
2. There are more people that I feel close to in this community	1	2	3	4
3. I know more people in this community well enough to say hello and have them say hello back	1	2	3	4
4. My motivation to look after my health has increased	1	2	3	4
5. I do more incidental activity e.g. take stairs, park further away etc	1	2	3	4
6. I eat more vegetables and/or salads	1	2	3	4
7. I consume less unhealthy foods and/or drinks	1	2	3	4

diabetes nsw & act

About the program

How likely is it that you would recommend this program to others? (0 = not at all likely and 10 = extremely likely)

0 1 2 3 4 5 6 7 8 9 10

NOT AT ALL LIKELY — EXTREMELY LIKELY

I was satisfied with the program:

1 No 2 Yes

How would you rate your facilitator/presenter?

1 Poor 2 Fair 3 Good 4 Very Good 5 Excellent

**Was this education session useful?**	Yes	No
Session 1: Physical activity for health	1	2
Session 2: Finding 'health' moments in your day	1	2
Session 3: Eat for health	1	2
Session 4: Bringing mindfulness into your daily practice	1	2

**Please choose one option for each statement:** **This program…**	Disagree ☹	Neutral 😐	Agree ☺
1. Was easy to understand	1	2	3
2. Suited my needs	1	2	3
3. Offered a supportive environment	1	2	3
4. Made me feel more connected to a supportive community	1	2	3

Thank you for taking the time to complete this survey.

DNSW & ACT MAY OCCASIONALLY USE SURVEY COMMENTS FOR PROMOTIONAL MATERIALS SO THAT OTHERS MAY BETTER UNDERSTAND THE BENEFITS OF ATTENDING. INFORMATION COLLECTED WITHIN THIS SURVEY IS COVERED BY THE DNSW & ACT PRIVACY POLICY. FOR MORE INFORMATION VISIT DIABETESNSW.COM.AU/PRIVACY/ OR CALL 1300 342 238.
